# Acute Mesenteric Ischemia as a Severe Complication Associated With Tirzepatide: A Case Report and Safety Alert

**DOI:** 10.7759/cureus.97035

**Published:** 2025-11-17

**Authors:** Shani Brooks, Samraiz Nafees, Khaled Abdi, Isobel Austin, Balal Mahmood, Adam Bowden, Emily Warren, Alexander Crossley, Azhar Mehmood, Victoria Birkett

**Affiliations:** 1 Emergency Medicine, York and Scarborough Teaching Hospitals NHS Foundation Trust, Scarborough, GBR; 2 Internal Medicine, York and Scarborough Teaching Hospitals NHS Foundation Trust, Scarborough, GBR; 3 Radiology, York and Scarborough Teaching Hospitals NHS Foundation Trust, Scarborough, GBR

**Keywords:** acute mesenteric ischemia, gastrointestinal complications, glp-1 receptor agonist, tirzepatide, unsupervised drug use, weight loss medication

## Abstract

Tirzepatide is a glucose-dependent insulinotropic polypeptide (GIP) and glucagon-like peptide-1 (GLP-1) receptor agonist used in the management of type 2 diabetes mellitus. We report a case of acute mesenteric ischemia in a patient following treatment with tirzepatide, highlighting a rare but serious adverse event potentially related to its effects on gastrointestinal motility and vascular perfusion. This case emphasizes the necessity for careful patient selection and close clinical monitoring, especially given the increasing use of this medication outside of formal supervision.

## Introduction

Tirzepatide, marketed under the brand name Mounjaro, is a dual glucose-dependent insulinotropic polypeptide (GIP) and glucagon-like peptide-1 (GLP-1) receptor agonist that was authorized for use in type 2 diabetes in May 2022 [1]. Since then, tirzepatide has been extensively evaluated in the SURPASS clinical trials and deemed safe for use in patients with diabetes [2]. A systematic review and meta-analysis in 2023 concluded that “Tirzepatide has significant potential as a weight loss drug, with little increase in adverse events compared with other weight loss drugs” [3]. Mounjaro was authorized for weight management in the United Kingdom on November 8, 2023 [4] and is now widely available for purchase online. The British National Formulary (BNF) states that tirzepatide is indicated for weight management “in conjunction with dietary measures and increased physical activity in individuals with a body mass index (BMI) of 30 kg/m² or more, or in individuals with a BMI of 27 kg/m² or more in the presence of at least one weight-related comorbidity” [5,6].

In clinical use, the most common drug‑related adverse effects of tirzepatide involve the gastrointestinal system, including nausea, vomiting, diarrhea, and delayed gastric emptying. Rare but notable complications such as pancreatitis, gallbladder disease, and bowel obstruction have also been observed [7,8]. Beyond these established effects, its potential impact on gastrointestinal vascular integrity remains less understood. In addition, other risk factors such as dehydration, hypotension, underlying atherosclerosis, or concurrent use of vasoconstrictive agents may further predispose individuals to gut ischemia. This case contributes to emerging evidence by describing a severe gastrointestinal complication associated with tirzepatide.

## Case presentation

A 61-year-old woman with a medical history notable for obesity (body mass index: 36.5 kg/m²), hiatus hernia, asthma, osteoarthritis, and hypertension presented acutely unwell. Her regular medications included omeprazole 40 mg daily, atorvastatin 20 mg daily, and inhaled salbutamol and beclomethasone for asthma management. One week prior to presentation, she had self-administered low-dose tirzepatide (2.5 mg subcutaneously once weekly) for weight loss, obtained online without medical supervision.

Approximately four hours after receiving her second dose of tirzepatide, she developed severe, diffuse abdominal pain accompanied by nausea, vomiting, and diarrhea. On arrival to the emergency department, she was tachycardic and hypotensive, with physical examination revealing generalized abdominal tenderness.

Laboratory investigations demonstrated leukocytosis (white cell count: 18.1 × 10^9^/L) with neutrophilia (17.08 × 10^9^/L) and an elevated lactate level, raising suspicion for ischemic bowel injury (Table 1). Stool cultures taken at admission were subsequently negative.

**Table 1:** Table showing raised white cell count and neutrophils indicating inflammation, a normal CRP, and markedly elevated lactate reflecting tissue hypoxia, all consistent with acute mesenteric ischemia and severe systemic response WCC: white cell count, CRP: C-reactive protein

Laboratory parameter	Patient’s result	Normal range
WCC	18.1 × 10^9^/L	4-11 × 10^9^/L
Neutrophils	17.08 × 10^9^/L	2-8 × 10^9^/L
CRP	4 mg/L	0-5 mg/L
Lactate	4.8 mmol/L	0.5-2.2 mmol/L

**Figure 1:**
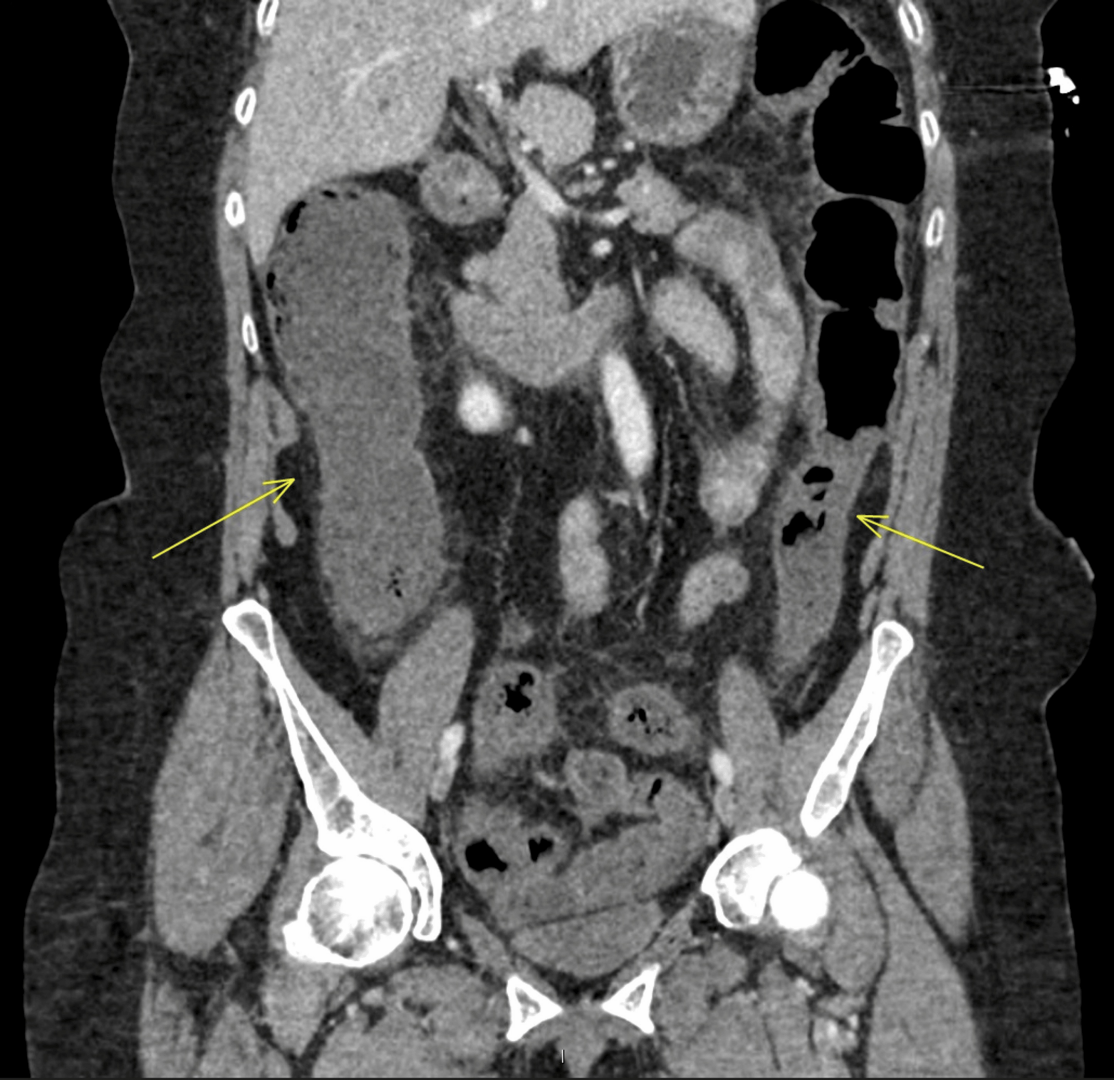
Coronal view of abdominal CT scan showing fat stranding and dilated colon with reduced wall enhancement (arrows) CT: computed tomography

**Figure 2:**
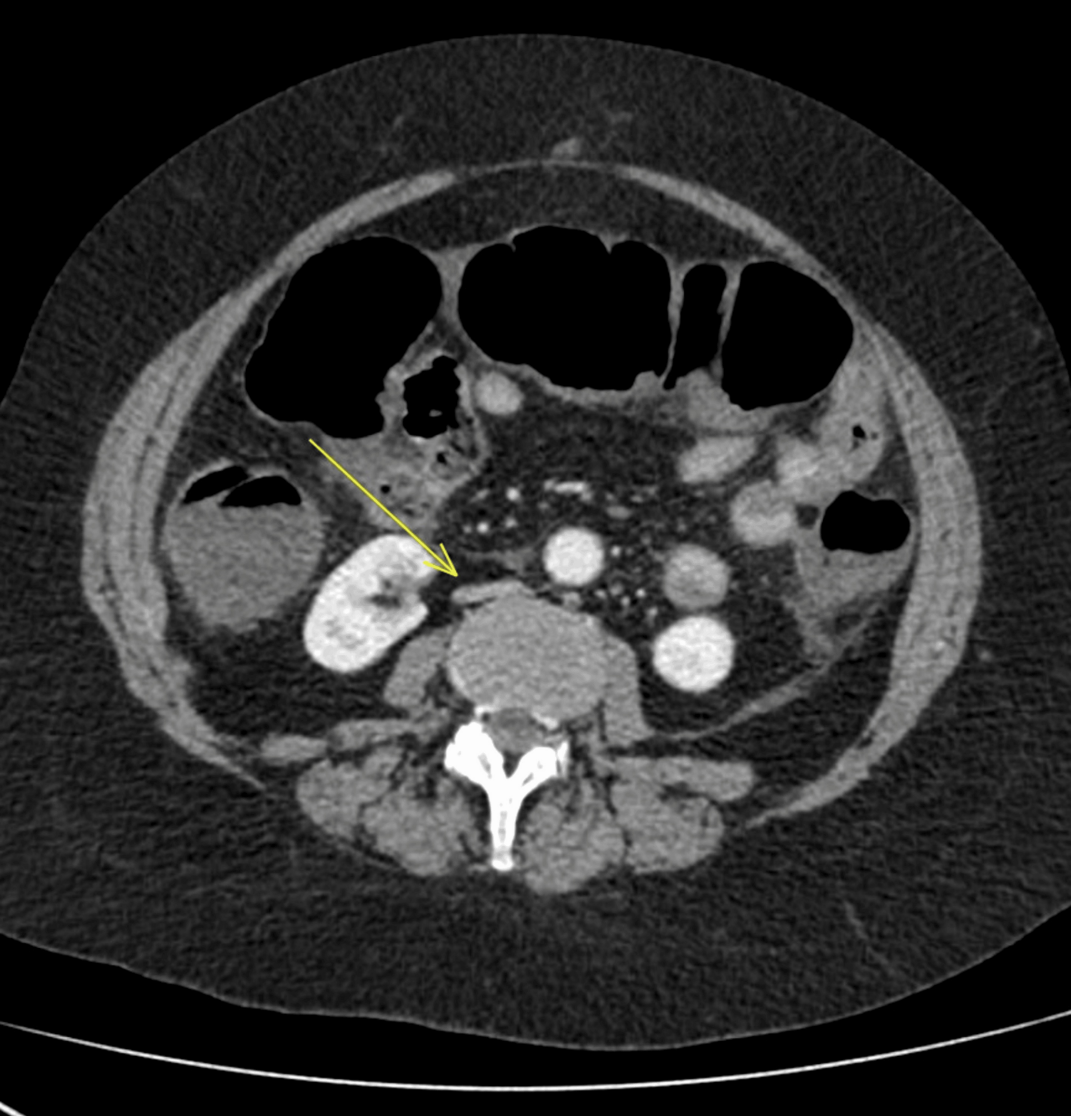
Axial view of abdominal CT showing IVC compression (arrow) and dilated large bowel loops CT: computed tomography, IVC: inferior vena cava

**Figure 3:**
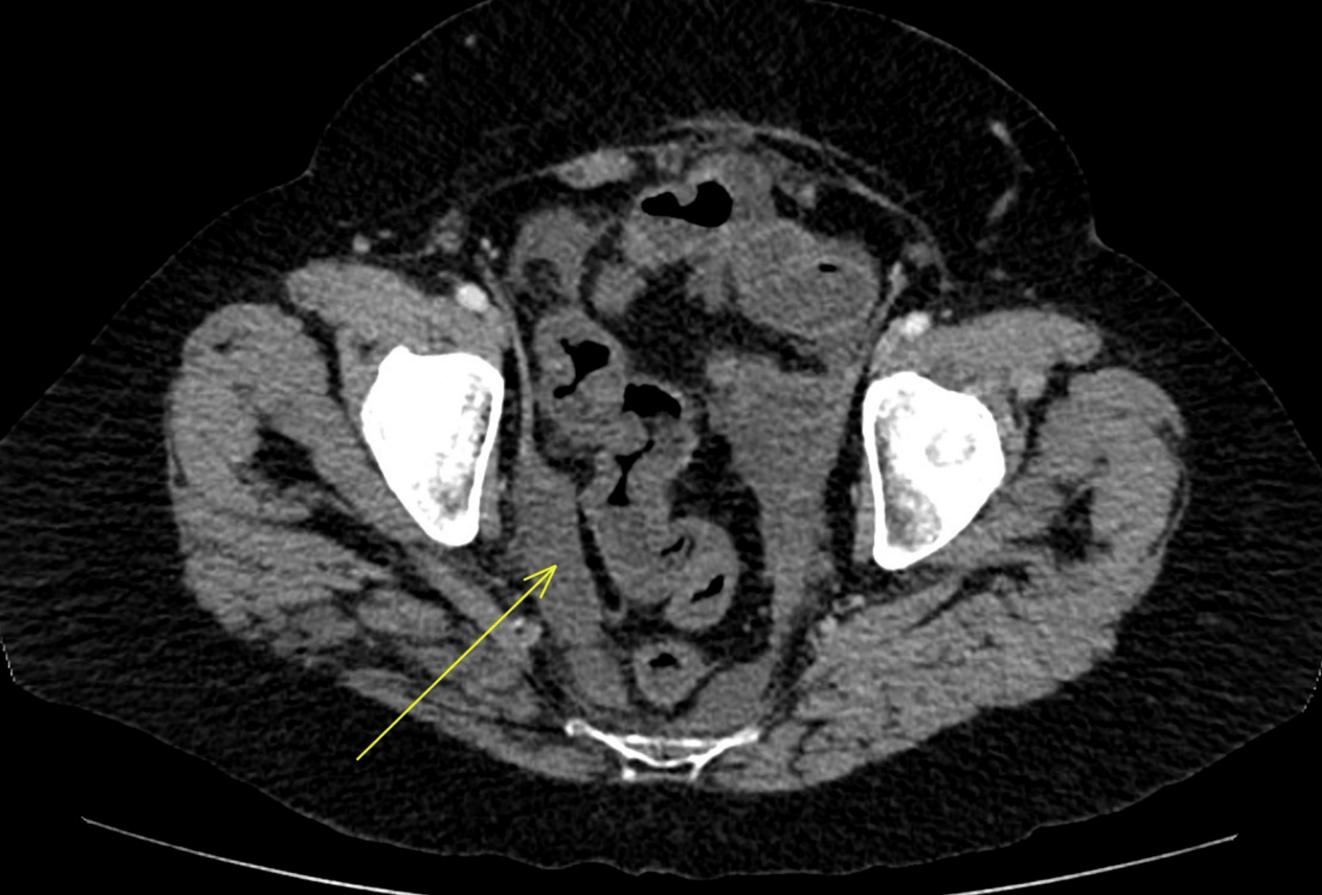
Axial view of abdominal CT showing increased bowel wall thickness, bowel mucosal enhancement, and ascites (arrow) CT: computed tomography

Contrast-enhanced computed tomography (CT) of the abdomen revealed moderate ascites, mild distension of the proximal large bowel, and a collapsed inferior vena cava (IVC) (Figures 1-3). The radiologist noted that early features of bowel ischemia could not be excluded. No signs of pancreatitis were identified, and differential diagnoses included inflammatory or infectious colitis.

Her clinical condition rapidly deteriorated, and 9.5 hours after presentation, she was taken to the operating theater. Upon induction of anesthesia, she was peri-arrest, with a systolic blood pressure of 48 mmHg and mottled skin, necessitating immediate resuscitation prior to surgery.

Intraoperative findings confirmed segmental bowel infarction requiring surgical resection. Histopathological examination revealed focal mucosal necrosis, edema, and congestion of the bowel wall, with mild ischemic changes extending to the resection margins, indicative of a widespread ischemic process. No evidence of thrombosis, embolism, or neoplasia was identified.

Tragically, despite intensive care support, she died before a planned second-look laparotomy. The cause of death was documented as “spontaneous acute small bowel ischemia.”

## Discussion

GLP-1 receptor agonists, including tirzepatide, affect gastrointestinal motility and perfusion, potentially leading to adverse events such as gastroparesis and intestinal hypoperfusion. Delayed gastric emptying may predispose individuals to increased intra-abdominal pressure, altered blood flow, and, in rare cases, ischemic injury. Additionally, GIP influences splanchnic circulation, further implicating a vascular mechanism in this case.

Similar cases have been reported, including those by Bayless et al., describing tirzepatide-associated colonic ischemia [9]. Furthermore, recent Medicines and Healthcare Products Regulatory Agency (MHRA) warnings highlight gastrointestinal complications associated with GLP-1 receptor agonists [10]. A review of clinical trial data suggests a potential link between these agents and rare but serious gastrointestinal vascular events.

This case also raises concerns about the increasing availability of tirzepatide online without medical oversight. Patients using such medications without appropriate counselling may fail to recognize early warning signs of ischemia, such as persistent abdominal pain and rising lactate levels.

## Conclusions

This case highlights the importance for clinicians to recognize the potential for serious gastrointestinal vascular complications associated with tirzepatide, including the rare but life-threatening possibility of mesenteric ischemia. Patients presenting with unexplained abdominal pain while receiving GLP-1 receptor agonists should be promptly evaluated for ischemic events to enable early diagnosis and intervention.

The increasing availability of tirzepatide through online sources highlights a pressing need for stricter regulatory measures to prevent unsupervised use and to ensure patient safety. Ultimately, further research is imperative to clarify the incidence, underlying mechanisms, and risk factors for bowel ischemia in patients treated with GLP-1 receptor agonists, so that clinicians can better identify vulnerable individuals and mitigate potentially fatal complications.

## References

[1] Tall Bull S, Nuffer W, Trujillo JM (2022). Tirzepatide: a novel, first-in-class, dual GIP/GLP-1 receptor agonist. J Diabetes Complications.

[2] Rosenstock J, Wysham C, Frías JP (2021). Efficacy and safety of a novel dual GIP and GLP-1 receptor agonist tirzepatide in patients with type 2 diabetes (SURPASS-1): a double-blind, randomised, phase 3 trial. Lancet.

[3] Tan B, Pan XH, Chew HS (2023). Efficacy and safety of tirzepatide for treatment of overweight or obesity. A systematic review and meta-analysis. Int J Obes (Lond).

[4] (2025). MHRA authorises diabetes drug Mounjaro (tirzepatide) for weight management and weight loss. https://www.gov.uk/government/news/mhra-authorises-diabetes-drug-mounjaro-tirzepatide-for-weight-management-and-weight-loss.

[5] Joint Formulary Committee (2025). Tirzepatide. https://bnf.nice.org.uk/drugs/tirzepatide/.

[6] Garvey WT, Frias JP, Jastreboff AM (2023). Tirzepatide once weekly for the treatment of obesity in people with type 2 diabetes (SURMOUNT-2): a double-blind, randomised, multicentre, placebo-controlled, phase 3 trial. Lancet.

[7] Mathew A, Hannoodee H (2023). FRI643 tirzepatide associated partial small bowel obstruction: a case report. J Endocr Soc.

[8] Gordon A, Dixit K, Shachi T, Salonia J, Seltzer E (2023). A rare case of a large bowel obstruction due to tirzepatide. Chest.

[9] Bayless D, Singh J, Park BU, Sweetser S (2024). Tirzepatide-associated colonic ischemia. ACG Case Rep J.

[10] (2025). GLP-1 receptor agonists: reminder of the potential side effects and to be aware of the potential for misuse. https://www.gov.uk/drug-safety-update/glp-1-receptor-agonists-reminder-of-the-potential-side-effects-and-to-be-aware-of-the-potential-for-misuse.

